# Effect of leukocytes on semen quality in men from primary and secondary infertile couples: A cross‐sectional study

**DOI:** 10.1002/hsr2.1683

**Published:** 2023-11-08

**Authors:** Shiwei Fan, Yuanqi Zhao, Zeling Zhang, Huiru Wang, Yifu Hou, Shun Bai, Ran Liu, Bo Xu

**Affiliations:** ^1^ Reproductive and Genetic Hospital, The First Affiliated Hospital of USTC, Division of Life Sciences and Medicine University of Science and Technology of China Hefei Anhui China; ^2^ Wannan Medical College Wuhu Anhui China; ^3^ Center for Reproductive Medicine Traditional Chinese Hospital of LuAn Lu'an Anhui China

**Keywords:** leukocytospermia, primary infertility, secondary infertility, semen quality

## Abstract

**Background and Aims:**

Leukocytospermia (LCS) is a known cause of male infertility. However, the relationship between seminal leukocytes and semen quality among infertile couples remains controversial. This study aims to investigate the association between semen quality and LCS in male partners of infertile couples.

**Methods:**

Semen samples were collected from 512 men who asked for a fertility evaluation in a reproductive center in China. Seminal leukocytes were counted following peroxidase staining with benzidine. Other semen parameters were compared in subfertile men with and without LCS.

**Results:**

Poor semen quality (e.g., low semen volume, sperm concentration, and sperm progressive/total motility) was observed among men with LCS compared to those without LCS. Men with LCS had a higher risk of low sperm progressive motility (OR = 0.99, 95% CI = 0.98−0.99, *p* = 0.02) and total motility (OR = 0.99, 95% CI = 0.98−0.99, *p* = 0.02), even after adjustment for potential confounders (both OR = 0.99, 95% CI = 0.98−0.99, *p* = 0.03). Lower sperm viability was observed in LCS from male partners of secondary couples, while no significant difference in semen parameters was found between men with and without LCS in male partners of primary infertile couples. Low sperm motility and viability were associated with LCS in men from secondary infertile couples after adjusting for confounders (OR = 0.97, 95% CI = 0.95−0.99, *p* = 0.04; OR = 0.94, 95% CI = 0.89−0.99, *p* = 0.04, respectively).

**Conclusions:**

Our findings indicate that a higher risk of abnormal semen parameters was correlated with an increased number of leukocytes in men from secondary infertile couples.

## 1 INTRODUCTION

Leukocytospermia (LCS), also known as leukospermia or pyospermia, is defined as a concentration of more than 1 × 10^6^/mL white blood cells (WBCs) in semen by the World Health Organization.^1^ Not only a true infection or an inflammatory process but also noninfectious causes, including toxins, varicocele, autoimmune disorders, chronic prostatitis, and congenital genitourinary malformations, can induce LCS.^2^ The prevalence of LCS is approximately 30% in infertile men with or without seminal bacterial infection.^3^ In semen, the majority of leukocytes are granulocytes (50%−60%), macrophages (20%−30%), and T lymphocytes (2%−5%).^4^, ^5^, ^6^ Notably, leukocytes stimulate the production of inflammatory mediators and cytokines, including reactive oxygen species (ROS) and nitric oxide.^7^, ^8^ High levels of ROS may cause a decline in testis injury, resulting in abnormal spermatogenesis and male infertility.^9^

The concentration of seminal WBCs was higher in infertile men than in fertile men.^10^, ^11^ However, the correlation between seminal leukocytes and sperm quality remains controversial. Several studies found that LCS was associated with abnormal semen parameters, including a reduction in sperm motility.^12^, ^13^ A positive correlation between LCS and sperm tail defects was reported by Aziz et al.^14^ Additionally, increased levels of ROS generated by leukocytes in semen induce lipid peroxidation and membrane damage, thereby impairing sperm motility and the acrosome reaction.^15^, ^16^ However, a systematic review with meta‐analysis of case‒control studies reported that LCS was not associated with reduced fertility after assisted reproductive technology and with altered semen quality in subfertile men seeking fertility evaluation.^17^

Infertility patterns are divided into primary (inability to become pregnant in married couples after at least 1 year of having unprotected sex) and secondary (inability to become pregnant in married couples after at least one pregnancy) types.^18^ A meta‐analysis including 21 studies showed that the prevalence of primary and secondary infertility in Africa was 49.9% and 49.7%, respectively.^19^ In addition to Africa, the proportion of secondary infertility increased in China among infertile couples from 2012 to 2016, thereby indicating that secondary infertility‐related factors need exploration in further studies.^20^ A large cohort of European primary infertile men study reported that the prevalence of LCS was 25%.^21^ Primary infertile men with LCS had lower sperm motility when compared to those men with non‐LCS.^22^ Given that the impact of inflammation increases over time, inflammation production by leukocytes may have an increasingly negative impact on semen quality in men with secondary infertility, meaning that poor lifestyle habits (e.g., smoking and excessive drinking) are more commonly associated with secondary than primary infertility. In addition, our previous study found seminal tract infection (STI) was associated with increased semen leukocyte counts in secondary infertile men but not in primary infertile men, suggesting semen leukocyte is strongly correlation with STI pathogens in secondary infertility.^23^ However, the potential relationship between leukocytes and semen parameters among men from primary and secondary infertile couples remains unclear. In this context, the aim of this study was to evaluate the association between seminal leukocytes and semen quality in men from subfertile couples.

## 2 METHODS

### 2.1 Participants

A total of 512 men who sought a fertility evaluation at the Reproductive Center of The First Affiliated Hospital of University of Science and Technology of China (USTC) from July 2021 to May 2022 were included in this study. All participants were asked for education and history of sexual and infections. Participants were enrolled according to the following criteria: (1) aged  ≥20 years; (2) without a child; (3) without symptomatic for genitourinary infections or inflammation. The exclusion criteria were men who were diagnosed with genetic defects related to the reproductive tract (e.g., mutation in azoospermic factor region or spermatogenesis‐associated genes), azoospermia, testicular trauma, cryptorchidism, and postmumps orchitis. This study was approved by The First Affiliated Hospital of USTC Ethical Committee (No. 2021‐RE‐072).

### 2.2 Semen analysis

Semen parameters (e.g., semen volume, sperm concentration, motility, and morphology) were determined according to the WHO criteria.^1^ Specimens were obtained after 2−7 days of sexual abstinence. Semen parameters were evaluated after liquefaction at 37°C for at least 30 min. Sperm concentration and motility were determined by computer‐assisted sperm analysis (SAS). Sperm morphology was assessed by Diff‐Quick staining (Anke Biotechnology) and observed under a light microscope (CX33; Olympus Corporation). Antisperm antibodies (AsAs) were detected by the mixed antiglobulin reaction method (Anke Biotechnology). The sperm DNA fragmentation index (DFI) and high DNA stainability (HDS) were assessed by sperm chromatin structure assay (Celula). Leukocytes were detected by peroxidase staining (Anke Biotechnology).

### 2.3 Statistical analysis

Qualitative variables were described as frequencies (percentages), and quantitative variables were described as the mean ± standard deviation if normally distributed and medians (interquartile range) if not. Pearson's *χ* ^2^ test and Student's *t*‐test were used for parametric comparisons, and the Mann−Whitney *U* test was utilized for nonparametric comparisons. Logistic regression was used to examine the association between LCS and semen parameters. Sperm concentration was transformed by the natural logarithms to obtain normally distributed residuals and homoscedasticity. Covariates initially included age, BMI, smoking, and alcohol intake. A *p* value < 0.05 was considered to indicate statistical significance. All statistical analyses were two‐sided and performed using Prism 9.0.

## 3 RESULTS

The clinical characteristics and semen parameters of 512 subjects are presented in Table 1. The age and BMI of the subjects were 31.1 ± 4.7 years and 24.8 ± 3.9 kg/m^2^, respectively. Lifestyle factors, including smoking and alcohol intake, were found in 41.0% and 52.5% of subjects, respectively. A total of 51.2% of subjects were recruited from infertile couples, of which 170 subjects (64.9%) were from primary infertile couples and the remaining 92 subjects (35.1%) were from secondary infertile couples.

**Table 1:** Clinical characteristics and semen parameters of the whole cohort.

Clinical characteristics	Total (*n* = 512)	LCS (*n* = 202)	Non‐LCS (*n* = 310)	*p*
Age (years), mean ± SD	31.1 ± 4.7	31.5 ± 4.7	30.8 ± 4.7	0.09
BMI (kg/m^2^), mean ± SD	24.8 ± 3.9	24.7 ± 4.1	24.8 ± 3.7	0.79
Education, *n* (%)				0.17
Junior high school or less	99 (19.3)	43 (21.3)	56 (18.0)	
High school	93 (18.2)	41 (20.3)	52 (16.8)	
College/university	320 (62.5)	118 (58.4)	202 (65.2)	
Smoking status, *n* (%)				0.09
Nonsmoker	302 (59.0)	110 (54.5)	192 (61.9)	
Smoker	210 (41.0)	92 (45.5)	118 (38.1)	
Alcohol status, *n* (%)				0.98
Nondrinker	243 (47.5)	96 (47.5)	147 (47.4)	
Drinker	269 (52.5)	106 (52.5)	163 (52.6)	
Infertility, *n* (%)	262 (51.2)	104 (51.5)	158 (51.0)	0.91
Infertility types, *n* (%)				0.69
Primary infertility, *n* (%)	170 (64.9)	69 (66.3)	101 (63.9)	
Secondary infertility, *n* (%)	92 (35.1)	35 (33.7)	57 (36.1)	
Semen parameters (median [IQR])				
Abstinence time (days)	4.0 (3.0−7.0)	4.0 (3.0−6.0)	4.0 (3.0−7.0)	0.22
Semen volume (mL)	3.1 (2.3−4.2)	2.9 (2.2−3.9)	3.4 (2.4−4.3)	0.02
Sperm concentration (**×**10^6^/mL)	59.2 (31.9−109.5)	52.3 (27.8−98.2)	65.8 (33.9−113.4)	0.04
Progressive motility (%)	38.9 (25.6−50.7)	36.6 (21.8−49.0)	40.0 (27.2−52.0)	0.03
Total motility (%)	43.4 (29.5−57.2)	41.1 (26.4−55.7)	44.7 (31.8−58.6)	0.02
Normal morphology (%)	7.0 (5.0−9.0)	7.0 (5.0−8.0)	7.0 (5.0−9.0)	0.16
Leukocytes (×10^6^/mL)	0.25 (0.04−1.62)	2.06 (1.35−4.63)	0.05 (0.03−0.16)	<0.001
AsAs (%)	1.0 (1.0−3.0) (*n* = 306)	1.0 (1.0−3.0) (*n* = 116)	1.0 (1.0−3.0) (*n* = 190)	0.55
Viability (%)	74.0 (60.0−82.0) (*n* = 243)	72.0 (58.0−80.0) (*n* = 91)	74.0 (62.0−83.5) (*n* = 152)	0.23
DFI (%)	10.7 (6.5−14.6) (*n* = 96)	11.3 (6.7−15.3) (*n* = 42)	10.2 (6.4−13.8) (*n* = 54)	0.37
HDS (%)	6.8 (5.1−9.8) (*n* = 96)	7.5 (5.6−10.3) (*n* = 42)	6.6 (5.0−8.9) (*n* = 54)	0.16

*Note*: *p* Values were derived from Pearson's *χ* ^2^ test and Student's *t*‐test for parametric comparisons and the Mann−Whitney *U* test for nonparametric comparisons.

Abbreviations: AsAs, antisperm antibodies; BMI, body mass index; DFI, DNA fragmentation index; HDS, high DNA stainability; IQR, interquartile range; LCS, leukocytospermia; SD, standard deviation.

The studied population included 202 subjects with LCS and 310 subjects without LCS. Clinical characteristics (e.g., age, BMI, education, smoking, alcohol status, and infertility types) were similar in men with LCS and without LCS. However, poor semen quality (e.g., low semen volume, sperm concentration, and sperm progressive/total motility) was observed among men with LCS compared to those without LCS.

Table 2 shows the association between LCS and semen parameters (e.g., semen volume, sperm concentration, sperm progressive/total motility, sperm normal morphology, AsAs, sperm viability, sperm DFI, and HDS) by logistic regression analysis. Men with LCS had a higher risk of low sperm progressive motility (OR = 0.99, 95% CI = 0.98−0.99, *p* = 0.02) and total motility (OR = 0.99, 95% CI = 0.98−0.99, *p* = 0.02), even after adjustment for potential confounders including age, BMI, smoking, and drinking (both OR = 0.99, 95% CI = 0.98−0.99, *p* = 0.03).

**Table 2:** Logistic regression analyses for LCS and semen parameters.

	Crude		Adjusted	
Semen parameters	OR (95% CI)	*p*	OR (95% CI)	*p*
Semen volume	0.90 (0.79−1.01)	0.07	0.90 (0.80−1.02)	0.10
Sperm concentration	0.68 (0.44−1.04)	0.08	0.68 (0.44−1.05)	0.08
Progressive motility	0.99 (0.98−0.99)	0.02	0.99 (0.98−0.99)	0.03
Total motility	0.99 (0.98−0.99)	0.02	0.99 (0.98−0.99)	0.03
Normal morphology	0.95 (0.89−1.01)	0.13	0.95 (0.89−1.02)	0.14
AsAs	0.99 (0.95−1.03)	0.74	0.99 (0.95−1.03)	0.67
Viability	0.98 (0.97−1.00)	0.12	0.99 (0.97−1.00)	0.09
DFI	1.05 (0.98−1.12)	0.18	1.04 (0.97−1.12)	0.26
HDS	1.06 (0.95−1.18)	0.29	1.08 (0.96−1.21)	0.20

*Note*: Adjusted model, adjusted for age, BMI, smoking, and drinking.

Abbreviation: LCS, leukocytospermia.

The clinical characteristics and semen parameters of men from couples with primary and secondary infertility with and without LCS are summarized in Tables 3 and 4, respectively. Among male partners of primary infertile couples, no significant difference in semen parameters was found between men with and without LCS (Table 3). However, lower sperm viability was observed in LCS from male partners of secondary couples (Table 4). Similarly, other semen parameters (e.g., sperm concentration, sperm progressive, and total motility) were lower in men from secondary infertile couples with LCS than in those without LCS, although the differences were not statistically significant (Table 4).

**Table 3:** Clinical characteristics and semen parameters in men from primary infertile couples with LCS and without LCS.

Clinical characteristics		Primary infertile (*n* = 170)		
	Total	LCS (*n* = 69)	Non‐LCS (*n* = 101)	*p*
Age (years), mean ± SD	30.9 ± 4.3	31.1 ± 3.8	30.8 ± 4.6	0.65
BMI (kg/m^2^), mean ± SD	25.2 ± 4.2	24.8 ± 4.3	25.5 ± 4.0	0.30
Education, *n* (%)				0.18
Junior high school or less	30 (17.6)	14 (20.3)	16 (15.8)	
High school	37 (21.8)	18 (26.1)	19 (18.8)	
College/university	103 (60.6)	37 (53.6)	66 (65.3)	
Smoking status, *n* (%)				0.75
Nonsmoker	101 (59.4)	40 (58.0)	61 (60.4)	
Smoker	69 (40.6)	29 (42.0)	40 (39.6)	
Alcohol status, *n* (%)				0.83
Nondrinker	83 (48.8)	33 (47.8)	50 (49.5)	
Drinker	87 (51.2)	36 (52.2)	51 (50.5)	
Semen parameters (median [IQR])				
Abstinence time (days)	4.0 (3.0−7.0)	4.0 (2.5−6.0)	4.0 (3.0−7.0)	0.20
Semen volume (mL)	3.1 (2.2−4.2)	2.7 (2.1−3.9)	3.4 (2.3−4.3)	0.14
Sperm concentration (**×**10^6^/mL)	60.6 (28.3−117.5)	52.2 (28.7−101.7)	66.2 (26.0−125.9)	0.20
Progressive motility (%)	35.2 (24.1−49.0)	35.1 (20.5−49.2)	35.4 (26.1−49.0)	0.67
Total motility (%)	39.5 (28.2−54.6)	39.5 (26.0−56.6)	39.5 (29.5−54.1)	0.72
Normal morphology (%)	7.0 (5.0−9.0)	7.0 (5.0−8.0)	7.0 (5.0−9.0)	0.38
Leukocytes (×10^6^/mL)	0.25 (0.04−1.59)	2.09 (1.30−4.44)	0.05 (0.03−0.15)	<0.001
AsAs (%)	1.0 (1.0−3.0)	1.0 (0−3.0) (*n* = 38)	1.0 (1.0−2.3) (*n* = 62)	0.40
Viability (%)	70.0 (56.0−82.0)	70.0 (54.0−82.0) (*n* = 31)	70.0 (58.0−82.0) (*n* = 47)	0.96
DFI (%)	13.6 (7.9−16.7)	14.7 (10.0−23.1) (*n* = 14)	12.6 (6.2−15.9) (*n* = 12)	0.16
HDS (%)	8.5 (6.2−10.5)	9.0 (6.6−10.3) (*n* = 14)	6.7 (4.3−11.7) (*n* = 12)	0.35

*Note*: *p* Values were derived from Pearson's *χ* ^2^ test and Student's *t*‐test for parametric comparisons and the Mann−Whitney *U* test for nonparametric comparisons.

Abbreviations: AsAs, antisperm antibodies; BMI, body mass index; DFI, DNA fragmentation index; HDS, high DNA stainability; IQR, interquartile range; LCS, leukocytospermia; SD, standard deviation.

**Table 4:** Clinical characteristics and semen parameters in men from secondary infertile couples with LCS and without LCS.

Clinical characteristics		Secondary infertile (*n* = 92)		
	Total	LCS (*n* = 35)	Non‐LCS (*n* = 57)	*p*
Age (years), mean ± SD	33.3 ± 4.9	33.7 ± 4.5	33.1 ± 5.1	0.59
BMI (kg/m^2^), mean ± SD	24.9 ± 3.5	25.4 ± 2.9	24.5 ± 3.8	0.25
Education, *n* (%)				0.79
Junior high school or less	23 (25.0)	10 (28.6)	13 (22.8)	
High school	17 (18.5)	5 (14.3)	12 (21.1)	
College/university	52 (56.5)	20 (57.1)	32 (56.1)	
Smoking status, *n* (%)				0.89
Nonsmoker	57 (62.0)	22 (62.9)	35 (61.4)	
Smoker	35 (38.0)	13 (37.1)	22 (38.6)	
Alcohol status, *n* (%)				0.49
Nondrinker	41 (44.6)	14 (40.0)	27 (47.4)	
Drinker	51 (55.4)	21 (60.0)	30 (52.6)	
Semen parameters (median [IQR])				
Abstinence time (days)	4.0 (3.0−7.0)	4.0 (3.0−7.0)	4.0 (3.0−6.5)	0.97
Semen volume (mL)	3.5 (2.6−4.7)	3.0 (2.3−4.8)	3.6 (2.8−4.6)	0.29
Sperm concentration (**×**10^6^/mL)	60.8 (31.6−114.4)	44.1 (25.6−74.6)	67.9 (33.9−119.5)	0.09
Progressive motility (%)	38.2 (24.5−48.8)	32.5 (18.9−46.6)	42.0 (27.0−49.4)	0.09
Total motility (%)	44.0 (28.7−55.1)	37.3 (21.3−54.1)	47.6 (32.2−56.2)	0.09
Normal morphology (%)	7.0 (5.0−9.0)	7.0 (4.0−8.0)	7.0 (5.0−9.0)	0.29
Leukocytes (×10^6^/mL)	0.26 (0.04−1.56)	1.66 (1.36−2.46)	0.06 (0.03−0.24)	<0.001
AsAs (%)	2.0 (1.0−3.0) (*n* = 49)	1.0 (0.8−3.0) (*n* = 18)	2.0 (1.0−3.0) (*n* = 31)	0.51
Viability (%)	74.0 (64.5−82.0) (*n* = 36)	67.5 (49.5−73.5) (*n* = 12)	80.0 (68.0−84.0) (*n* = 24)	0.006
DFI (%)	10.3 (6.4−14.4) (*n* = 32)	10.7 (7.4−14.7) (*n* = 15)	8.0 (5.3−14.2) (*n* = 17)	0.58
HDS (%)	6.0 (5.1−8.1) (*n* = 32)	6.2 (5.6−10.1) (*n* = 15)	5.7 (4.6−7.7) (*n* = 17)	0.39

*Note*: *p* Values were derived from Pearson's *χ* ^2^ test and Student's *t*‐test for parametric comparisons and the Mann−Whitney *U* test for nonparametric comparisons.

Abbreviations: AsAs, antisperm antibodies; BMI, body mass index; DFI, DNA fragmentation index; HDS, high DNA stainability; IQR, interquartile range; LCS, leukocytospermia; SD, standard deviation.

The relationship between the LCS and semen parameters in men from couples with primary and secondary infertility was further investigated (Tables 5 and 6). Low sperm motility and viability were associated with LCS in men from secondary infertile couples after adjusting for confounders (OR = 0.97, 95% CI = 0.95−0.99, *p* = 0.04; OR = 0.94, 95% CI = 0.89−0.99, *p* = 0.04, respectively), whereas no associations between LCS and semen quality were found in male partners of primary infertile couples by either the crude or adjusted logistic regression model analysis.

**Table 5:** Logistic regression analyses for LCS and semen parameters in men from primary infertile couples.

	Crude		Adjusted	
Semen parameters	OR (95% CI)	*p*	OR (95% CI)	*p*
Semen volume	0.90 (0.72−1.10)	0.31	0.89 (0.71−1.11)	0.32
Sperm concentration	0.72 (0.37−1.37)	0.31	0.67 (0.34−1.30)	0.24
Progressive motility	0.99 (0.98−1.01)	0.71	0.99 (0.98−1.01)	0.66
Total motility	0.99 (0.98−1.01)	0.79	0.99 (0.98−1.01)	0.74
Normal morphology	0.95 (0.85−1.06)	0.36	0.94 (0.84−1.06)	0.32
AsAs	0.95 (0.82−1.08)	0.47	0.93 (0.79−1.07)	0.32
Viability	0.99 (0.97−1.02)	0.67	0.99 (0.97−1.03)	0.82
DFI	1.12 (0.99−1.32)	0.12	1.18 (1.00−1.47)	0.07
HDS	1.05 (0.88−1.27)	0.61	1.10 (0.89−1.40)	0.39

*Note*: Adjusted model, adjusted for age, BMI, smoking, and drinking.

Abbreviation: LCS, leukocytospermia.

**Table 6:** Logistic regression analyses for LCS and semen parameters in men from primary infertile couples.

	Crude		Adjusted	
Semen parameters	OR (95% CI)	*p*	OR (95% CI)	*p*
Semen volume	0.90 (0.68−1.17)	0.43	0.89 (0.67−1.17)	0.42
Sperm concentration	0.56 (0.19−1.61)	0.29	0.44 (0.14−1.34)	0.15
Progressive motility	0.98 (0.95−1.00)	0.07	0.97 (0.94−1.00)	0.05
Total motility	0.98 (0.95−1.00)	0.06	0.97 (0.95−0.99)	0.04
Normal morphology	0.93 (0.80−1.08)	0.37	0.92 (0.78−1.07)	0.30
AsAs	0.98 (0.79−1.17)	0.80	1.00 (0.80−1.20)	0.99
Viability	0.95 (0.89−1.00)	0.06	0.94 (0.89−0.99)	0.04
DFI	1.01 (0.89−1.14)	0.87	1.05 (0.91−1.21)	0.51
HDS	1.12 (0.88−1.48)	0.38	1.30 (0.94−1.84)	0.12

*Note*: Adjusted model, adjusted for age, BMI, smoking, and drinking.

Abbreviation: LCS, leukocytospermia.

## 4 DISCUSSION

This study examined the potential association of LCS with semen parameters among men from infertile couples. We observed that men with LCS exhibited poorer semen quality than those without LCS in male partners of secondary infertile couples rather than in male partners of primary infertile couples.

Semen contains not only male germ cells but also nongerm cells, including epithelial cells and leukocytes.^24^ Several investigations have reported that increased leukocyte counts are related to poor semen quality and male infertility.^2^, ^6^, ^8^, ^25^ Recently, a meta‐analysis of 28 case‒controlled retrospective studies including 1320 LCS and 4856 non‐LCS provided comprehensive information on the association between leukocytes and semen parameters.^17^ The results showed that the sperm progressive motility was significantly lower in leukocytospermic samples than in nonleukocytospermic samples when leukocytes were detected by peroxidase test. In current study, compared with the men from infertile couples without LCS, men with LCS had significantly decreased semen volume, sperm concentration, progressive motility, and total motility. These data are similar to our previous study that reported poor semen quality (e.g., low semen volume, sperm concentration, and sperm progressive/total motility) was observed among men with LCS compared to those with non‐LCS.^12^ Abnormal semen parameters in men with LCS may be due to high levels of ROS production in semen by activated leukocytes.^26^

Previousstudies have investigated the mechanisms involved in the relationship between LCS and semen quality.^8^, ^27^ It is well known that increased leukocyte counts in semen are associated with oxidative stress and elevated levels of inflammatory cytokines.^28^ Importantly, inflammatory cytokines recruit and activate leukocytes, which results in increased ROS production.^29^ Previous studies have reported that sperm quality was decreased, especially low sperm motility, after incubation of spermatozoa with several cytokines, including IL‐6, TNF‐α, IFN‐γ, IL‐17A, and IL‐1β.^30^, ^31^, ^32^ Thus, inflammatory cytokines released by leukocytes may lead to an inflammatory response resulting in poor semen quality.

It is worth noting that several studies have reported that leukocytes do not play a critical role in men undergoing fertility evaluation.^17^ However, the majority of the studies do not separate the infertility types (primary infertility and secondary infertility).^17^, ^33^, ^34^ In terms of factors that cause infertility, inflammation by elevated seminal leukocyte counts is a chronic process. Unlike other factors such as genetic defects or absence of vas deferens that induce severe oligoasthenoterazoospermia or azoospermia, men with LCS may have a minor impact on semen parameters and the risk of pregnancy. Accordingly, we speculated that the effect of LCS in men from secondary infertile couples was higher than that in men from primary infertile couples. Our data showed trends toward an adverse correlation between semen quality and LCS in male partners of secondary infertile couples, although there was no difference in the incidence of LCS among men from primary and secondary infertile couples. Among several semen parameters, sperm motility and viability in LCS were more likely to be lower than those in non‐LCS in male partners of secondary infertile couples, indicating that leukocyte‐related oxidative stress contributes to sperm apoptosis. Thus, LCS showed a decreasing trend in sperm concentration and sperm motility in men from secondary infertile couples.

The major strength of our study was the assessment of the association between semen quality and LCS in men from different infertile couples. In addition, several potential confounders, including age, BMI, drinking, and smoking, were also evaluated in this study. Since the correlation between leukocytes and semen parameters is still unclear, this study further advances LCS in men from secondary infertile couples and should be taken into consideration as a factor to assess semen quality.

However, the present study also had some limitations. A single center‐based cross‐sectional study increases the possible selection bias. Additionally, seminal leukocyte detection by the peroxidase method only identifies granulocytes, thereby resulting in an underestimation of the association of macrophages and lymphocytes with semen quality. Furthermore, a direct correlation between LCS and clinical pregnant is not include in this study. However, it is worth noting that granulocytes are the major subtype of leukocytes in seminal fluid; therefore, the limitation in the assay for leukocytes is a relatively minor contribution.

In this cross‐sectional study, we observed negative associations of increased seminal leukocyte counts with semen quality in men from secondary infertile couples as measured by lower sperm viability. The findings indicated that a higher risk of abnormal semen parameters was correlated with an increased number of leukocytes in men from secondary infertile couples, whereas in men from primary infertile couples, the results need to be confirmed in large prospective cohort studies.

## AUTHOR CONTRIBUTIONS

**Shiwei Fan**: Conceptualization; data curation; resources; software. **Yuanqi Zhao**: Data curation; investigation; resources; software; visualization. **Zeling Zhang**: Data curation; formal analysis; investigation; methodology; software. **Huiru Wang**: Data curation; investigation; methodology; software. **Yifu Hou**: Data curation; formal analysis; investigation. **Shun Bai**: Conceptualization; data curation; investigation; methodology; project administration; writing—original draft. **Ran Liu**: Data curation; methodology; project administration; software; writing—review and editing. **Bo Xu**: Conceptualization; project administration; writing—review and editing. All authors have read and approved the final version of the manuscript.

## CONFLICT OF INTEREST STATEMENT

The authors declare no conflict of interest.

## ETHICS STATEMENT

This study was approved by The First Affiliated Hospital of USTC Ethical Committee (No. 2021‐RE‐072). Written informed consent was obtained from all participants.

## TRANSPARENCY STATEMENT

The lead author Shun Bai, Ran Liu, Bo Xu affirms that this manuscript is an honest, accurate, and transparent account of the study being reported; that no important aspects of the study have been omitted; and that any discrepancies from the study as planned (and, if relevant, registered) have been explained.

## Data Availability

All data are available from the corresponding author upon reasonable request. The corresponding author had full access to all of the data in this study and takes complete responsibility for the integrity of the data and the accuracy of the data analysis.
